# Posterior Corneal Characteristics of Cataract Patients with High Myopia

**DOI:** 10.1371/journal.pone.0162012

**Published:** 2016-09-07

**Authors:** Qinghe Jing, Yating Tang, Dongjin Qian, Yi Lu, Yongxiang Jiang

**Affiliations:** 1 Department of Ophthalmology and Vision Science, Eye and ENT Hospital of Fudan University, Shanghai, China; 2 Key Laboratory of Myopia of State Health Ministry, and Key Laboratory of Visual Impairment and Restoration of Shanghai, Shanghai, China; Xiamen University, CHINA

## Abstract

**Purpose:**

To evaluate the characteristics of the posterior corneal surface in patients with high myopia before cataract surgery.

**Methods:**

We performed a cross-sectional study at the Eye and ENT Hospital of Fudan University, Shanghai, China. Corneal astigmatism and axial length were measured with a rotating Scheimpflug camera (Pentacam) and partial coherence interferometry (IOLMaster) in a high-myopia study group of 167 eyes (axial length ≥ 26 mm) and a control group of 150 eyes (axial length > 20 mm and < 25 mm).

**Results:**

Total corneal astigmatism and anterior corneal astigmatism values were higher in the high-myopia group than in the control group. There was no significant difference in posterior corneal astigmatism between the high-myopia study group and the control group. In the study group, the mean posterior corneal astigmatism (range 0 – −0.9 diopters) was –0.29 diopters (D) ± 0.17 standard deviations (SD). The steep corneal meridian was aligned vertically (60°–120°) in 87.43% of eyes for the posterior corneal surface, and did not change with increasing age. There was a significant correlation (r = 0.235, p = 0.002) between posterior corneal astigmatism and anterior corneal astigmatism, especially when the anterior corneal surface showed with-the-rule (WTR) astigmatism (r = 0.452, p = 0.000). There was a weak negative correlation between posterior corneal astigmatism and age (r = –0.15, p = 0.053) in the high-myopia group. Compared with total corneal astigmatism values, the anterior corneal measurements alone overestimated WTR astigmatism by a mean of 0.27 ± 0.18 D in 68.75% of eyes, underestimated against-the-rule (ATR) astigmatism by a mean of 0.41 ± 0.28 D in 88.89% of eyes, and underestimated oblique astigmatism by a mean of 0.24 ± 0.13 D in 63.64% of eyes.

**Conclusions:**

Posterior corneal astigmatism decreased with age and remained as ATR astigmatism in most cases of high myopia. There was a significant correlation between posterior corneal astigmatism and anterior corneal astigmatism when anterior corneal astigmatism was WTR. If posterior corneal astigmatism is not accounted for when selecting toric intraocular lenses for high-myopia patients, the use of anterior corneal astigmatism measurements alone will lead to overestimation of WTR astigmatism and underestimation of ATR and oblique astigmatism.

## Introduction

Myopia is the most common human eye disorder worldwide, affecting 85%–90% of young adults in Asian countries such as Singapore and Taiwan [1]. Of rural Chinese, 26.7% of those ≥ 30 years old and 22.9% of those ≥ 40 years old are affected [2,3]. In China, the prevalence of high myopia, defined as eyes with spherical equivalent refractive error of at least −6.00 D and axial length of at least 26.0 mm [4], is ~1.3%–26% [2,3]. In 2004, Tuft and Bunce [5] found an association between increased axial length and lower mean age of cataract surgery. As there is an increasing prevalence of cataract surgery in younger age groups, it is important to understand the relationship between high myopia and cataracts. Pan et al. [6] confirmed an association between myopia and nuclear and posterior subcapsular (PSC) cataracts. Many cataract patients with high myopia also have astigmatism, and treatment with a toric intraocular lens (IOL) has been effective in patients with high myopic astigmatism [7,8]. However, previous methods of calculating toric IOL power that are based on anterior corneal astigmatism measurements have led to residual astigmatism and refractive errors in a small percentage of patients [9]. As total corneal astigmatism includes both anterior and posterior corneal astigmatism, the total corneal astigmatism cannot be optimally calculated with anterior corneal astigmatism measurements and the keratometric index alone. Previous studies have reported a mean magnitude of posterior corneal astigmatism of −0.30 D [10]. Furthermore, the anterior corneal surface shifted from with-the-rule (WTR) to against-the-rule (ATR) astigmatism with age, whereas the posterior corneal surface continued to show ATR astigmatism in most cases [10–16]. Therefore, patient-specific posterior corneal astigmatism measurements are required to estimate total corneal astigmatism. As the characteristics of corneal astigmatism in high myopia remain unknown, we performed a cross-sectional study of anterior and posterior corneal astigmatism measurements in high-myopia patients and compared these values with those obtained in controls.

We measured the axial length using an IOLMaster (Carl Zeiss Meditec, Jena, Germany). We measured the corneal parameters using a Pentacam (OCULUS, Wetzlar, Germany), which uses a rotating Scheimpflug camera to image the anterior segment (including both the anterior and posterior corneal surfaces). It measures 25,000 data points from 50 meridians across the cornea in less than 2 seconds, which is more accurate than Scheimpflug photography. This device can automatically start the scan when correct alignment and focus with the patient’s cornea has been achieved [17], which is convenient for the examiner. Finally, the Pentacam shows good repeatability of corneal curvature measurements, reported in diopters, not only anteriorly but also posteriorly [18].

## Methods

In this cross-sectional study, we recruited patients scheduled for cataract surgery at the Eye and ENT Hospital of Fudan University, Shanghai, China, between September 1 and December 31, 2014. Patients were classified into two groups, cataract patients with high myopia and cataract patients without myopia. Axial length and corneal biometric data were collected from all patients. Patients with a history of previous ocular surgery, corneal disease, uveitis, glaucoma, wearing contact lenses within the previous 2 weeks, or age younger than 40 years were excluded. The study was approved by the Human Research Ethics Committee of the Eye and ENT Hospital of Fudan University and adhered to the tenets of the Declaration of Helsinki. Written informed consent was obtained from each patient.

All measurements were performed in the auto mode by two experienced examiners, therefore eliminating any potential examiner bias. Patients were examined one or more times until the data were accepted as accurate within the error limits of the instruments. The obtained data included the mean keratometric mid-radius of curvature in diopters (Km), central corneal thickness (CCT), total corneal astigmatism, anterior corneal astigmatism, and posterior corneal astigmatism. Total corneal astigmatism was calculated using a proprietary method of merging the Scheimpflug data of the anterior and posterior cornea.

WTR astigmatism was defined as a cylindrical error for a steep corneal meridian between 90° ± 30°, and ATR astigmatism was defined as a cylindrical error for a steep corneal meridian between 0° ± 30°. All other astigmatic measurements outside these parameters were termed oblique astigmatism. These definitions applied to astigmatism of the total corneal and anterior corneal surfaces. For the posterior corneal surface, a steep corneal meridian between 90° ± 30° was considered ATR astigmatism and between 0° ± 30° was considered WTR astigmatism. To evaluate error in selecting toric IOLs in the clinical setting, based on anterior corneal astigmatism measurements alone, we calculated the difference between total corneal astigmatism and anterior corneal astigmatism.

To estimate the relationship between changes in corneal shape and axial length, we divided the high-myopia group into three subgroups according to axial length, as follows: patients with axial length ≥ 26 mm and < 28 mm; patients with axial length ≥ 28 mm and < 30 mm; and patients with axial length ≥ 30 mm.

Independent-sample t-test, χ^2^ test, and Fisher’s exact test were used to compare the astigmatism values and astigmatism types between the high-myopia and control groups. One-way analysis of variance, Kruskal–Wallis test, Pearson’s χ^2^ test, and Fisher’s exact test were used to compare the astigmatism values and astigmatism types among the age and axial-length subgroups in the high-myopia group. The relationships between posterior corneal astigmatism and anterior corneal astigmatism, age, axial length, and central corneal thickness were analyzed using Pearson’s correlation. Data analysis was performed using SPSS version 20.0 (SPSS, IBM Corp., Armonk, NY, USA). A p-value of < 0.05 was regarded as statistically significant.

## Results

The study included 317 eyes of 197 patients. There were 167 eyes of 98 patients in the high-myopia group and 150 eyes of 99 patients in the control group. The patient demographics are listed in Table 1. The Km values of the anterior and posterior corneal surfaces were lower in the high-myopia group than in the control group (Table 2). The magnitude of astigmatism of the total cornea and the anterior corneal surface was higher in the high-myopia study group than in the control group, whereas the posterior corneal astigmatism showed no statistically significant difference between the two groups. The anterior corneal surface astigmatism was mainly WTR in both groups (47.9% of eyes in the high-myopia group and 58% of eyes in the control group), whereas the posterior corneal surface astigmatism was mainly ATR in both groups (87.43% of eyes in the high-myopia group and 88% of eyes in the control group). In the high-myopia study group, there was no statistically significant difference for any corneal parameter between the axial-length subgroups (Table 3).

**Table 1:** Demographics of the study population.

		High myopia	Control	P value
**Eyes/patients**		167/98	150/99	
**Age(years)**	**Mean ± SD**	59.54±8.33	61.17±8.31	0.083^#^
	**Range**	41.82	41.82	
**Male/female**		76/91	66/84	0.787^##^
**Axial length**	**Mean ± SD**	29.41±2.43	23.42±0.88	0.000^#^
	**Range**	26.04,36.24	20.92,24.95	

SD = standard deviation.

^#^t-test

^##^Pearson’s χ^2^ test

**Table 2:** Corneal characteristics of the high-myopia and control groups.

		High myopia	Control	P value
**KmF (D)**	**Mean±SD**	43.18±1.83	43.82±1.5	0.001^#^
**KmB (D)**	**Mean±SD**	-6.32±0.27	-6.44±0.26	0.000^#^
**CCT (mm)**	**Mean±SD**	542.22±33.66	544.66±31.03	0.505^#^
**Astig. C (D)**	**Mean±SD**	1.06±0.70	0.89±0.67	0.022^#^
	**Range**	0.1, 4.2	0.1, 4.9	
**Astig. F (D)**	**Mean±SD**	0.99±0.67	0.84±0.68	0.044^#^
	**Range**	0.1, 4.4	0, 5.3	
**Astig. B (D)**	**Mean±SD**	-0.29±0.17	-0.28±0.16	0.627^#^
	**Range**	0, -0.9	0, -1.1	
**Diff. (D)**	**Mean±SD**	0.07±0.34	0.05±0.31	0.498^#^
	**Range**	-0.9, 1.5	-0.7, 1.1	
**Astig. type**	**WTR (C)**	66	64	0.846^##^
	**ATR (C)**	70	59	
	**Obl. (C)**	31	27	
	**WTR (F)**	80	87	0.199^##^
	**ATR (F)**	54	39	
	**Obl. (F)**	33	24	
	**No astig. (B)**	5	0	0.177^###^
	**WTR (B)**	10	12	
	**ATR (B)**	146	132	
	**Obl. (B)**	6	6	

Km = mean keratometry; CCT = central corneal thickness; Astig. = astigmatism value; Diff. = difference, Astig. C–Astig. F; Astig. type = astigmatism type; WTR = with the rule; ATR = against the rule; Obl. = oblique; No astig. = no astigmatism; D = diopters; C = total cornea; F = anterior corneal surface; B = posterior corneal surface; SD = standard deviation.

^#^t-test

^##^Pearson’s χ^2^ test

^###^Fisher’s exact test

**Table 3:** Corneal characteristics of high myopia by axial length.

Axial length group(mm)		26–28	28–30	≥30	P value
**n**		56	46	65	
**Axial length(mm)**	**Mean±SD**	26.82±0.51	28.98±0.62	31.95±1.50	0.000^#^
**Astig. C (D)**	**Mean±S**	0.96±0.62	1.13±0.81	1.11±0.69	0.500^#^
	**Range**	0.1, 4.2	0.1, 3.6	0.1, 3.7	
**Astig. F (D)**	**Mean±SD**	0.93±0.63	1.04±0.81	1.01±0.61	0.697^##^
	**Range**	0.1, 4.4	0.1, 4	0.1, 3.2	
**Astig. B (D)**	**Mean±SD**	-0.25±0.13	-0.32±0.21	-0.30±0.18	0.396^#^
	**Range**	0, -0.7	0, -0.9	-0.1, -0.9	
**Diff. (D)**	**Mean±SD**	0.03±0.20	0.09±0.39	0.10±0.39	0.404^#^
	**Range**	-0.4, 0.4	-0.6, 1.1	-0.9, 1.5	
**Astig. type**	**WTR (C)**	21	22	23	0.392^###^
	**ATR (C)**	21	18	31	
	**Obl. (C)**	14	6	11	
	**WTR (F)**	28	24	28	0.585^###^
	**ATR (F)**	15	16	23	
	**Obl. (F)**	13	6	14	
	**No astig. (B)**	3	2	0	0.126^####^
	**WTR (B)**	45	40	61	
	**ATR (B)**	2	3	1	
	**Obl. (B)**	6	1	3	

Astig. = astigmatism value; Diff. = difference, Astig. C–Astig. F; Astig. type = astigmatism type; WTR = with the rule; ATR = against the rule; Obl. = oblique; No astig. = no astigmatism; D = diopters; C = total cornea; F = anterior corneal surface; B = posterior corneal surface; SD = standard deviation.

^#^Kruskal–Wallis test

^##^one-way ANOVA

^###^Pearson’s χ^2^ test

^####^Fisher’s exact test

The study group was divided into three subgroups according to age: 40–50 years, 27 eyes; 51–60 years, 63 eyes; and ≥ 61 years, 77 eyes (Table 4). The mean posterior corneal astigmatism was higher in the 40–50 age group (–0.40 ± 0.20 D) than in the other two groups (–0.25 ± 0.15 D, 51–60 years; and –0.28 ± 0.17 D, ≥ 61 years). The difference between total corneal astigmatism and anterior corneal astigmatism in the different age groups was –0.11 ± 0.38 D, 40–50 years; 0.094 ± 0.28 D, 51–60 years; and 0.12 ± 0.35 D, ≥ 61 years (F = 5.137, p = 0.007). The main astigmatism type of the total cornea and anterior corneal surfaces in patients ≤ 50 years was WTR; in the other two age groups, however, there was a significantly higher percentage of eyes with ATR and oblique astigmatism, particularly for the total cornea. In contrast, the astigmatism type of the posterior corneal surface was stable among the age groups (Table 4).

**Table 4:** Corneal characteristics of high myopia by age.

Age group(year)		≤50	51–60	>61	P value
**n**		27	63	77	
**Axial length(mm)**	**Mean±SD**	29.49±2.62	29.67±2.51	29.17±2.30	0.475^#^
	**Range**	26.08, 33.89	26.09, 36.24	26.04, 34.15	
**Astig. C (D)**	**Mean±SD**	1.25±1.06	0.93±0.48	1.11±0.70	0.407^##^
	**Range**	0.1, 4.2	0.1, 2	0.1, 3.6	
**Astig. F (D)**	**Mean±SD**	1.36±1.00	0.84±0.47	0.99±0.63	0.057^##^
	**Range**	0.3, 4.4	0.1, 2.4	0.1, 4	
**Astig. B (D)**	**Mean±SD**	-0.40±0.20	-0.25±0.15	-0.28±0.17	0.001^#^
	**Range**	-0.1, -0.8	0, -0.8	0, -0.9	
**Diff. (D)**	**Mean±SD**	-0.11±0.38	0.094±0.28	0.12±0.35	0.007^#^
	**Range**	-0.6, 1.5	-0.7, 0.7	-0.9, 1.1	
**Astig. type**	**WTR (C)**	20	18	28	0.001^###^
	**ATR (C)**	4	33	33	
	**Obl. (C)**	3	12	16	
	**WTR (F)**	22	23	35	0.003^###^
	**ATR (F)**	3	24	27	
	**Obl. (F)**	2	16	15	
	**No astig. (B)**	0	3	2	0.621^####^
	**WTR (B)**	0	3	3	
	**ATR (B)**	27	52	67	
	**Obl. (B)**	0	5	5	

Astig. = astigmatism value; Diff. = difference, Astig. C–Astig. F; Astig. type = astigmatism type; WTR = with the rule; ATR = against the rule; Obl. = oblique; No astig. = no astigmatism; D = diopters; C = total cornea; F = anterior corneal surface; B = posterior corneal surface; SD = standard deviation.

^#^one-way ANOVA

^##^Kruskal–Wallis

^###^Pearson’s χ^2^ test

^####^Fisher’s exact test

**Fig 1:**
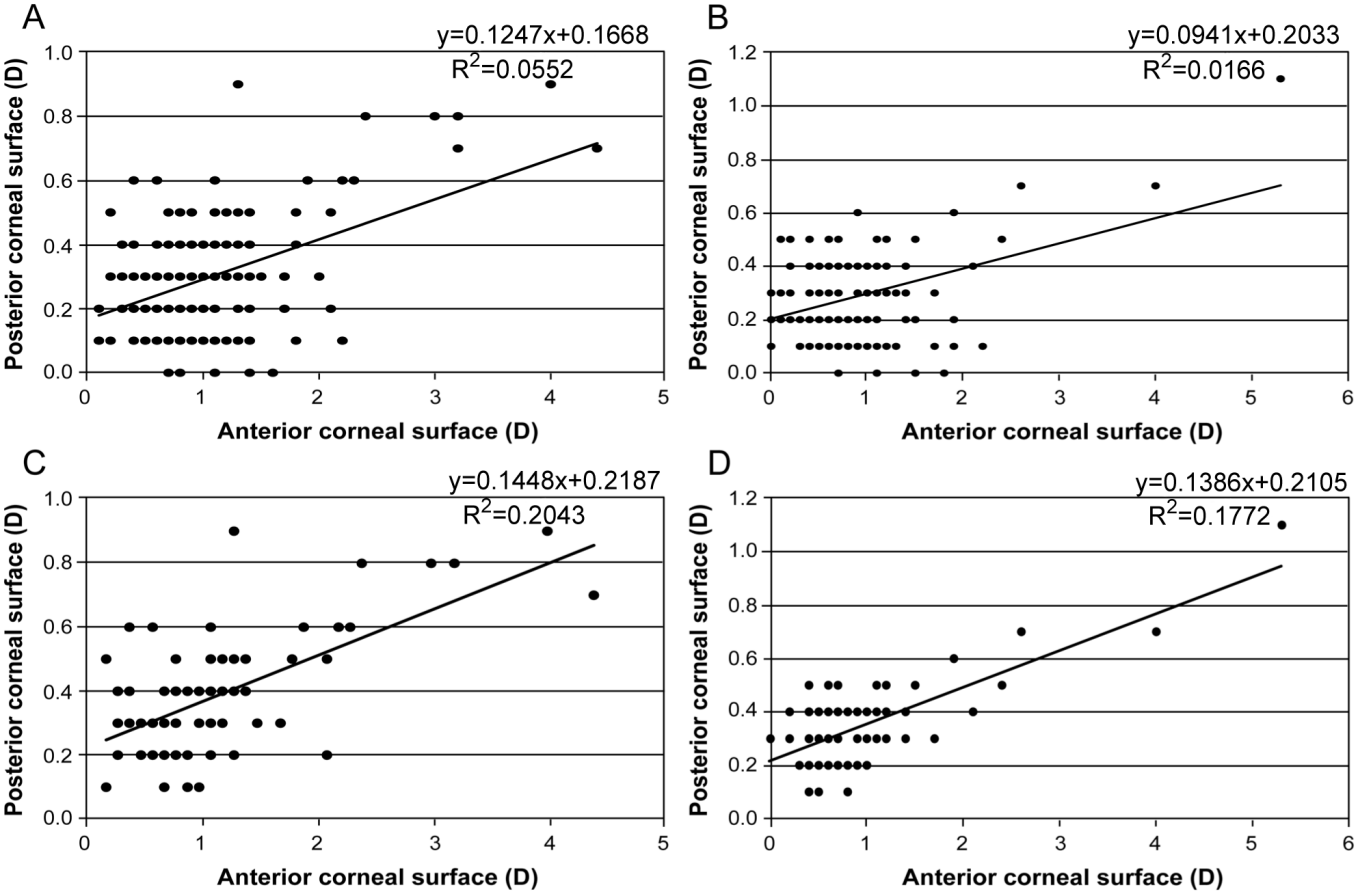
Correlation of the magnitude of astigmatism between posterior corneal surface and anterior corneal surface. A: high-myopia group (r = 0.235, p = 0.002), B: control group (r = 0.129, p = 0.115), C: anterior corneal surface with WTR astigmatism in the high-myopia group (r = 0.452, p = 0.000), D: anterior corneal surface with WTR astigmatism in the control group (r = 0.421, p = 0.000).

In the high-myopia group, there was a significant positive correlation (r = 0.235, p = 0.002) (Fig 1A) between posterior corneal astigmatism and anterior corneal astigmatism, especially when the steep meridian was within 90° ± 30° on the anterior corneal surface (r = 0.452, p = 0.000) (Fig 1C). In the control group, the same correlation was found between posterior corneal astigmatism and anterior corneal astigmatism when the anterior corneal surface had WTR astigmatism (r = 0.421, p = 0.000) (Fig 1D). However, the overall correlation between the magnitudes of anterior and posterior corneal astigmatism (r = 0.129, p = 0.115) was not significant in the control group (Fig 1B). There was a slight correlation between the values of posterior corneal astigmatism and age (r = –0.15, p = 0.053) in the high-myopia group. No significant correlation between the magnitude of posterior corneal astigmatism and total corneal astigmatism, axial length, or central corneal thickness was found between the two groups.

**Fig 2:**
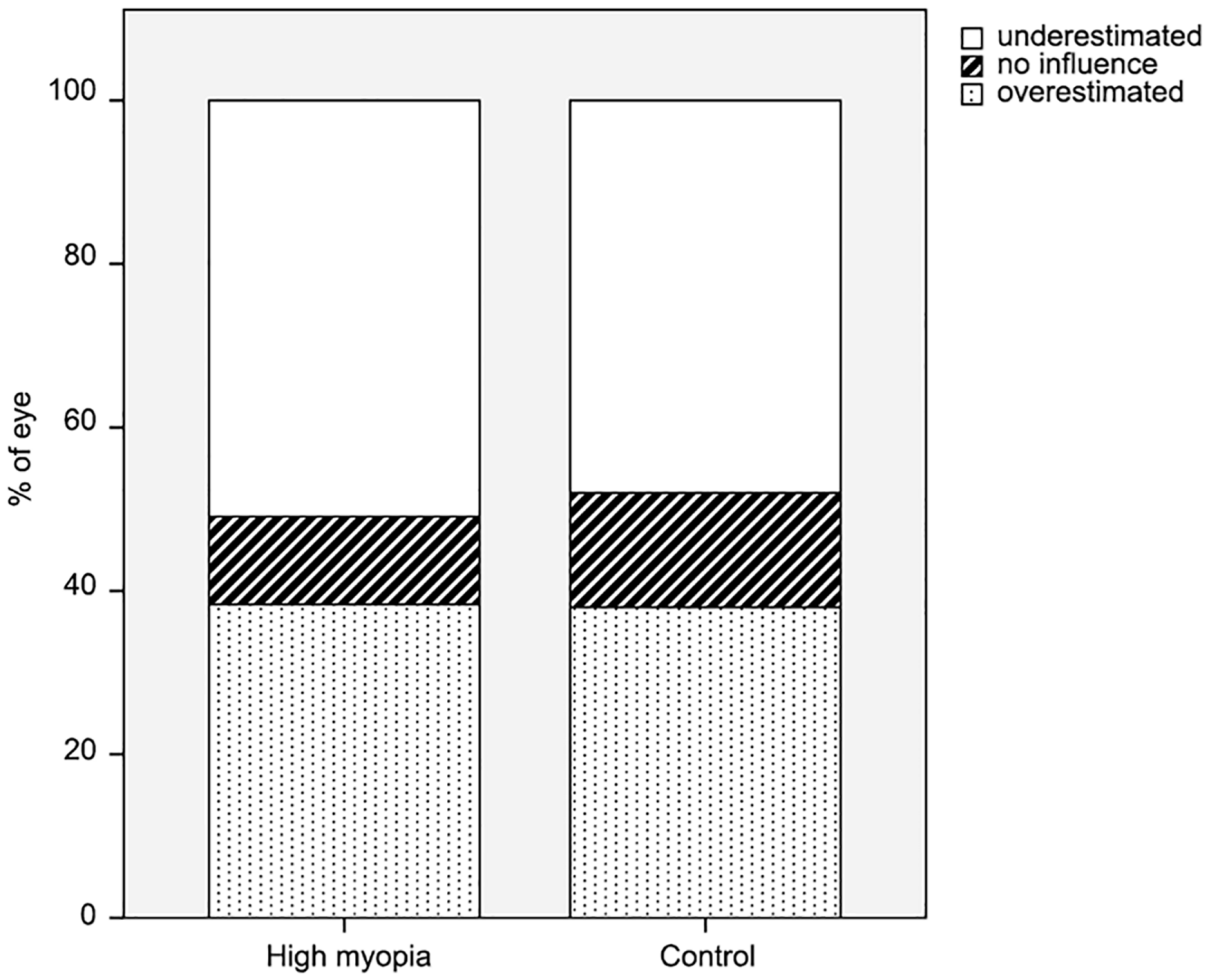
Estimation of total corneal astigmatism by anterior corneal measurement. No statistically significant difference in total corneal astigmatism was found between the high-myopia and control groups (p = 0.669).

Anterior corneal measurements underestimated total corneal astigmatism by 0.33 ± 0.24 D in 50.9% of eyes in the high-myopia study group and by 0.30 ± 0.22 D in 48% of eyes in the control group (t = 0.715, p = 0.476). Anterior corneal measurements overestimated total corneal astigmatism by 0.25 ± 0.18 D in 38.32% of eyes in the high-myopia group and by 0.26 ± 0.15 D in 38% of eyes in the control group (t = 0.313, p = 0.755). Fig 2 shows the percentage of eyes with different estimated astigmatism types between the groups. The difference between the magnitude of total corneal astigmatism and that of anterior corneal astigmatism in the high-myopia group was –0.14 ± 0.25 D, 0.36 ± 0.29 D, and 0.12 ± 0.19 D for WTR, ATR, and oblique astigmatism types, respectively. Compared with total corneal astigmatism values, anterior corneal measurements overestimated WTR astigmatism by a mean of 0.27 ± 0.18 D in 68.75% of the eyes, underestimated ATR astigmatism by a mean of 0.41 ± 0.28 D in 88.89% of the eyes, and underestimated oblique astigmatism by a mean of 0.24 ± 0.13 D in 63.64% of the eyes (Table 5).

**Table 5:** Anterior corneal measurement estimations of total corneal astigmatism by steepness of corneal meridian.

	Overestimated		Underestimated		No influence	Total
	n, %	Mean±SD (D)	n, %	Mean±SD (D)	n, %	n, %
**WTR**						
**±10°**	28, 71.79%	0.29±0.17	5, 12.82%	0.16±0.09	6, 15.38%	39, 100%
±20°	48, 73.85%	0.27±0.16	9, 13.85%	0.21±0.16	8, 12.31%	65, 100%
±30°	55, 68.75%	0.27±0.18	16, 20%	0.21±0.13	9, 11.25%	80, 100%
**ATR**						
**±10°**	1, 4.17%	0.1	22, 91.67%	0.5±0.35	1, 4.17%	24, 100%
±20°	1, 2.38%	0.1	39, 92.86%	0.43±0.30	2, 4.76%	42, 100%
±30°	1, 1.85%	0.1	48, 88.89%	0.41±0.28	5, 9.26%	54, 100%
**Obl**	8, 24.24%	0.13±0.05	21, 63.64%	0.24±0.13	4, 12.12%	33, 100%
**Total**	64	0.25±0.18	85	0.33±0.24	18	167, 100%

WTR = with the rule; ATR = against the rule; Obl. = oblique; SD = standard deviation.

WTR ± 10° = steep meridian of 80°–100°; WTR ± 20° = steep meridian of 70°–110°; WTR ± 30° = steep meridian of 60°–120°; ATR ± 10° = steep meridian of 0°–10° and 170°–180°; ATR ± 20° = steep meridian of 0°–20° and 160°–180°; ATR ± 30° = steep meridian of 0°–30° and 150°–180°; Obl. = steep meridian of 30°–60° and 120°–150°.

## Discussion

The study of corneal astigmatism is greatly enhanced by improvements in the accuracy of the measuring instruments. The mean magnitude of posterior corneal astigmatism is approximately –0.3 D and the steep posterior surface corneal meridian is aligned vertically in more than 85% of eyes [10,13–15]. However, the characteristics of posterior corneal astigmatism in high myopia are less well known. Our study found that in high myopia, the mean posterior corneal astigmatism was −0.29 ± 0.17 D. Furthermore, the posterior corneal steep meridian was aligned vertically in 87.43% of eyes and did not change with advancing age. There was a positive correlation between posterior corneal astigmatism and anterior corneal astigmatism, especially when the anterior corneal surface showed WTR astigmatism. Finally, anterior corneal measurements alone were inaccurate in estimating total corneal astigmatism.

The magnitude of astigmatism on the posterior corneal surface was similar between the high-myopia and control groups, which suggested that posterior corneal astigmatism was stable in high myopia. Kaye et al. [19] found that although there was a correlation between total astigmatism and myopia, corneal astigmatism and myopia were not related. In addition, vitreous depth is the major factor responsible for eye growth and the development of myopia [20,21], which may explain the similarity of the posterior corneal astigmatism values between the two groups in the present study.

The magnitude of posterior corneal astigmatism in the high-myopia group was –0.29 ± 0.17 D, which agrees well with the values recorded in normal eyes [10,13,16]. Among the different age groups, however, the mean posterior corneal astigmatism was higher in patients aged 40–50 years (–0.40 ± 0.20 D) than in the older two age groups (–0.25 ± 0.15 D, 51–60 years; and –0.28 ± 0.17 D, ≥ 61 years). This resulted in a weak negative correlation between the values of posterior corneal astigmatism and age (r = –0.15, p = 0.053) in the high-myopia group. It is unknown why posterior corneal astigmatism should decline with age.

The main astigmatism type of the total cornea and anterior corneal surfaces in the high-myopia group shifted from WTR astigmatism to ATR astigmatism with advancing age, whereas the astigmatism type of the posterior corneal surface was unchanged, as reported in previous studies of normal eyes [10,11,13–15]. The mechanisms proposed for the age-related shift in the astigmatism axis include reduction in lid tension, age-related changes in extraocular muscle tension, increased intraocular pressure, and changes to corneal structure [11].

The difference between total corneal astigmatism and anterior corneal astigmatism in the different age groups was –0.11 ± 0.38 D, 40–50 years; 0.094 ± 0.28 D, 51–60 years; and 0.12 ± 0.35 D, ≥61 years (Table 4; F = 5.137, p = 0.007). The average compensation effects progressively decline with age [11], a finding that our data upheld. A previous study [11], however, reported augmentation rather than compensation in the age groups of 61–70 and ≥ 71 years; in the present study, this finding was present in the 51–60 and ≥ 61 age groups. This shift in the different age groups is caused by ‘vector summation’, where the steep meridian of the anterior corneal surface shifts from vertical to horizontal with age, while the steep meridian of the posterior corneal surface is vertically stable in most cases.

In the high-myopia group, no statistically significant difference in any evaluated corneal parameter was found among the axial-length subgroups, indicating that axial length has no effect on corneal astigmatism. A previous study reported that as the level of myopia increased, axial length increased and the corneal radius of curvature decreased [20–23]. Furthermore, a significant positive correlation was found between corneal radius of curvature and axial length [23], not only in the flattest meridian, but also in the steepest meridian of the anterior corneal surface, and the changes in the two meridians were similar [24]. An association between astigmatism and axial length in high myopia has not been investigated previously. If change of the corneal radius of curvature with increased axial length is stable, the astigmatism will not change with increased axial length.

There was a significant positive correlation between posterior corneal astigmatism and anterior corneal astigmatism for the high myopia group, especially when the steep meridian was aligned vertically for the anterior corneal surface, which agreed well with the findings of previous studies [10,14]. However, we found no correlation between the magnitude of posterior corneal astigmatism and total corneal astigmatism. Although the steep meridian of the posterior cornea was relatively stable in most cases, the magnitude of posterior corneal astigmatism varied with changes in anterior corneal astigmatism in the high-myopia eyes. There are several possible reasons for the difference between the high-myopia and control groups with respect to the correlation between posterior and anterior corneal astigmatism. First, the magnitude of anterior corneal astigmatism was higher in the high-myopia group than in the control group (high-myopia group, 0.99 ± 0.67 D; control group, 0.84 ± 0.68 D; p = 0.044). Second, the distribution of anterior corneal astigmatism was wider in the control group than the high-myopia group (high-myopia group, 0.1–4.4 D; control group, 0–5.3 D). Finally, the small sample sizes and narrow age range in our study may have affected the results.

Although the present study was limited to patients > 40 years of age, our results provide new information regarding posterior and anterior corneal surface astigmatism in high-myopia eyes compared with normal eyes. In particular, the magnitude of posterior corneal astigmatism decreased with age but ATR astigmatism remained in most cases of high myopia. Establishing a correlation between posterior and anterior corneal astigmatism is useful if an instrument such as the Pentacam is not available for estimation of the magnitude of posterior corneal astigmatism. When such an instrument is available, however, measuring the anterior, posterior and total corneal astigmatism enables selection of the intraocular lens that will provide the optimal postoperative visual function for the patient.

Compared with total corneal astigmatism values, anterior corneal measurements overestimated WTR astigmatism by 0.14 ± 0.25 D, underestimated ATR astigmatism by 0.36 ± 0.29 D, and underestimated oblique astigmatism by 0.12 ± 0.19 D. Furthermore, anterior corneal measurements alone overestimated WTR astigmatism in 68.75% of eyes, underestimated ATR astigmatism in 88.89% of eyes, and underestimated oblique astigmatism in 63.64% of eyes. Therefore, measurements of anterior corneal astigmatism alone are less accurate for estimating total corneal astigmatism. To provide optimal visual correction in high-myopia patients, full measurement of corneal topography prior to implantation of a toric IOL enables identification of posterior corneal astigmatism, and is necessary to achieve the best correction of astigmatism.

There are two limitations in our study. First, there were fewer patients in the age 40–50 group than in the other two groups, although the number of eyes was >20. Second, the patients we chose were all over 40 years old, so these statistics are only for this age group. More research should be undertaken to analyze the characteristics of the posterior corneal surface for other age groups.

In conclusion, the magnitude of posterior corneal astigmatism tended to decrease with aging and remained ATR astigmatism in most cases of high myopia. Ignoring customized posterior corneal astigmatism will lead to overestimates of WTR astigmatism and underestimates of ATR and oblique astigmatism when selecting toric IOLs for high-myopia patients.
